# 2798. Difficult to treat GES and KPC harboring *Pseudomonas aeruginosa*: Evaluation of *in vivo* efficacy of ß-lactam/ß-lactamase inhibitor humanized exposure vs. *in vitro* efficacy

**DOI:** 10.1093/ofid/ofad500.2409

**Published:** 2023-11-27

**Authors:** Victor H Ruiz, Christian Gill, David P Nicolau

**Affiliations:** Harford Hospital Center for Anti-infective Research and Development, Hartford, Connecticut; Hartford Hospital Center for Anti-infective Research and Development, Hartford, Connecticut; Hartford Hospital, Hartford, Connecticut

## Abstract

**Background:**

GES and KPC harboring *Pseudomonas aeruginosa* (PSA) pose a challenge clinically as therapeutic alternatives are limited, and clinical data is scarce. Murine models provide translational data that can provide insight into therapeutic alternatives, and assist in clinical guidance. Herein, we evaluated the efficacy of human-simulated regimens (HSRs) of ceftazidime/avibactam (CZA), imipenem/relebactam (I/R), and meropenem/vaborbactam (MVB) in a murine thigh infection model to evaluate *in vivo* efficacy against serine-carbapenemase-producing PSA.

**Methods:**

9 PSA clinical isolates harboring GES 5 (n=1), GES 20 (n=1), GES 5/20 (n=1), GES 19/20 (n=3), and KPC (n=3) were evaluated. Groups of 6 mice were administered HSRs of CZA 2.5 g q8h as a 2h infusion (inf), I/R 1.25 g q6h as 0.5h inf, and MVB 4 g q8h as a 3h inf. Groups of 6 mice were sacrificed at 0h to determine initial bacterial burden (0h control) or treated with sham control and sacrificed at 24 hours to assess in vivo growth (24h control). Change in CFU/thigh relative to 0h was assessed and efficacy was defined as achieving ≥ 1-log_10_ kill at 24h relative to 0h. The percent of isolates meeting the 1-log_10_ kill endpoint were assessed.

**Results:**

Baseline bacterial burden was 5.2 ± 0.4 log_10_ CFU/thigh. Adequate growth was observed in control mice with ≥ 2 log_10_ CFU/thigh. CZA and I/R HSRs produced ≥ 1-log_10_ of kill against 100% and 83% of GES positive isolates, respectively (CZA MIC range 2- >64 mg/L; I/R MIC range 1- >32 mg/L). Activity was consistent against GES and KPC harboring isolates despite elevated *in vitro* MICs in some isolates. MVB activity was variable with only 44% of isolates reaching 1-log_10_ kill (MIC range 16 - >64). MVB failed in 67% of GES and 33% of KPC producing isolates all with elevated MICs. I/R and CZA *in vivo/ in vitro* discordance may be due to higher relebactam and avibactam concentrations achieved in the humanized exposure relative to the *in vitro* fixed concentrations.

**Conclusion:**

I/R and CZA were active against all KPC and GES harboring PSA isolates tested *in vivo*. The activity of MVB was variable suggesting this may be an inferior treatment option. Further studies to evaluate clinical outcomes in GES and KPC producing PSA using CZA and I/R are warranted given their increasing prevalence worldwide.

**Disclosures:**

**Christian Gill, PharmD**, Cepheid: Grant/Research Support|Entasis: Grant/Research Support|Everest Medicines: Grant/Research Support|Shionogi: Grant/Research Support **David P. Nicolau, PharmD**, Allergan: Advisor/Consultant|Allergan: Grant/Research Support|Cepheid: Advisor/Consultant|Cepheid: Grant/Research Support|Merck: Advisor/Consultant|Merck: Grant/Research Support|Pfizer: Advisor/Consultant|Pfizer: Grant/Research Support|Shionogi: Advisor/Consultant|Shionogi: Grant/Research Support|Tetraphase: Advisor/Consultant|Tetraphase: Grant/Research Support|Venatorx: Advisor/Consultant|Venatorx: Grant/Research Support|Wockhardt: Advisor/Consultant|Wockhardt: Grant/Research Support

